# Tuberculosis and COVID-19 Dually Affect Human Th17 Cell Immune Response

**DOI:** 10.3390/biomedicines11082123

**Published:** 2023-07-27

**Authors:** Anna Starshinova, Igor Kudryavtsev, Artem Rubinstein, Anna Malkova, Irina Dovgaluk, Dmitry Kudlay

**Affiliations:** 1 Almazov National Medical Research Centre, 197341 St-Petersburg, Russia; 2 Department of Immunology, Institution of Experimental Medicine, 197022 St-Petersburg, Russia; igorek1981@yandex.ru (I.K.); arrubin6@mail.ru (A.R.); 3 Faculty of Natural Sciences, Ariel University, Ariel 40700, Israel; anya.malkova.95@mail.ru; 4 Phthisiopulmonology Department, Research Institute of Phthisiopulmonology, 191036 St-Petersburg, Russia; prdovgaluk@mail.ru; 5 Department of Pharmacology, I.M. Sechenov First Moscow State Medical University, 119435 Moscow, Russia; d624254@gmail.com; 6 Institute of Immunology FMBA of Russia, 115478 Moscow, Russia

**Keywords:** tuberculosis, pulmonary tuberculosis, latent tuberculosis infection, coronavirus infection, COVID-19, SARS-CoV-2, autoimmunity, Treg, follicular Treg, Treg subsets

## Abstract

COVID-19 infection not only profoundly impacts the detection of tuberculosis infection (Tbc) but also affects modality in tuberculosis patient immune response. It is important to determine immune response alterations in latent tuberculosis infection as well as in SARS-CoV-2-infected tuberculosis patients. Such changes may have underlying effects on the development and course of further tuberculosis. Here, we aimed to review the characteristics of immune response in TB patients or convalescent COVID-19 patients with latent TB infection (LTBI). Materials and Methods. We analyzed the features of immune response in tuberculosis and COVID-19 patients. For this, we analyzed publications released from December 2019 to March 2023; those which were published in accessible international databases (“Medline”, “PubMed”, “Scopus”) and with keywords such as “COVID-19”, “SARS-CoV-2”, “tuberculosis”, “pulmonary tuberculosis”, “latent tuberculosis infection”, “Treg”, “follicular Treg”, and “Treg subsets”, we considered. Results. Through our analysis, we found that tuberculosis patients who had been infected with COVID-19 previously and elevated Th1 and Th2 cell levels. High levels of Th1 and Th2 cells may serve as a positive marker, characterizing activated immune response during TB infection. COVID-19 or post-COVID-19 subjects showed decreased Th17 levels, indicating a lack of tuberculosis development. Moreover, the typical course of tuberculosis is associated with an increase in Treg level, but COVID-19 contributes to a hyperinflammatory response. Conclusion. According to the data obtained, the course of tuberculosis proceeds in a dissimilar way due to the distinct immune response, elicited by SARS-CoV-2. Importantly, the development of active tuberculosis with a severe course is associated with a decline in Treg levels. Both pathogens lead to disturbed immune responses, increasing the risk of developing severe TB. The insights and findings of this paper may be used to improve the future management of individuals with latent and active tuberculosis.

## 1. Introduction

The rapid spread of SARS-CoV-2 (severe acute respiratory syndrome coronavirus 2), which induced a novel coronavirus (COVID-19) caused a pandemic and global public health concerns [1,2]. Currently, we know that COVID-19 potentiates the development of diverse pathological events by altering the immune status of patients [3,4,5]. In many countries around the world, the COVID-19 pandemic has had an impact on establishing approaches for the diagnosis, surveillance, and treatment of tuberculosis (TB) patients [6]. The WHO estimated that, mortality attributed to increased TB infection up to 1.5 million people (1.4 million people died from TB in 2019)) in 2021. At the same time, there has been an 18% decline in TB prevalence as evidenced by a decrease from 7.1 million cases in 2019 to 5.8 million in 2020 [7,8].

However, our focus as researchers is not solely on the potential issues, related to the diagnosis of TB and how it is able to influence increased morbidity and mortality in TB patients, as we believe the specificity of altered immune response in COVID-19 patients, displaying characteristics similar to that of the cellular immune response of TB patients is deserving of study [9,10,11]. The immunological processes understanding may provide a basis for developing new approaches for early TB diagnostics and development. Strikingly, extensive organ damage in COVID-19 occurs due to ACE2 (angiotensin-converting enzyme 2) expression [12,13], allowing SARS-CoV-2 to enter host cells due to several receptors and accessory proteins, including CD147, NRP-1, CD26, AGTR2, Band3, KREMEN1, ASGR1, ANP, TMEM30A, CLEC4G, and LDLRAD3, which promote entry into human cells. It should be noted that the altered characteristics of SARS-CoV-2 (B.1.1.7 (Alpha)) and its different variants—B.1.617.1 (Kappa), B.1.617.2 (Delta), B.1.617.2+ (Delta+), and B.1.1.529 (Omicron)—reflect changes in its ability to enter host cells [14,15].

Currently, receptors (CD147, ASGR1, etc.) are known to facilitate the entry of various viruses (and bacteria) such as measles, HIV, hepatitis B virus (HBV), hepatitis C virus (HCV), Ebola virus, etc. [16,17]. COVID-19, caused by SARS-CoV-2, has been shown to follow a seasonal trend, presented by varying symptoms and severity governed by the variant strain of SARS-CoV-2, as well as patient’s age, sex, underlying comorbidities, and individual immune status [18,19].

Most of the immune-mediated changes are associated with COVID-19 [19]. The suppression of proinflammatory mechanisms contributes to virus replication, resulting in potentiated pyroptosis, an inflammatory form of programmed cell death typical of cytopathic viral infections [20]. At the same time, there is an elevated release of necrosis products that enhance inflammation due to pyroptosis products and the development of an aberrant inflammatory response [21].

Moreover, some virus strains are responsible for the upregulated surface expression of MHC molecules on infected cells that are able to promote autoimmune response in virus-infected lung cells. COVID-19 augments MHC expression and promotes autoimmune responses; however, this remains poorly understood [21].

Immunity suppression is found in TB patients, which can be aggravated during or after COVID-19 [22] and deteriorate disease course, resulting in developing severe pulmonary fibrosis and antibacterial resistance. Obtaining new data on the pathogenesis of fibrosis, as well as immunologic and autoimmune alterations in individuals with LTBI and TB after COVID-19, may be a key in predicting TB course and adjusting therapeutic protocols [23]. 

At the same time, recent evidence suggests that SARS-CoV-2 may affect TB pathogenesis and its clinical course. Despite the recency effect of the COVID-19 pandemic, a comprehensive body of research on the innate and adaptive immune responses and molecular mechanisms underlying SARS-CoV-2 entry, as well as its spread in human cells, has accumulated. Here, we aimed to identify SARS-CoV-2-driven alterations in immune response in patients with LTBI and TB patients. Similarities and differences in immune cell phenotype, driven by both pathogens, have been uncovered. It should be of importance for the prognosis behind TB and its prophylaxis.

## 2. Methods 

**Figure 1:**
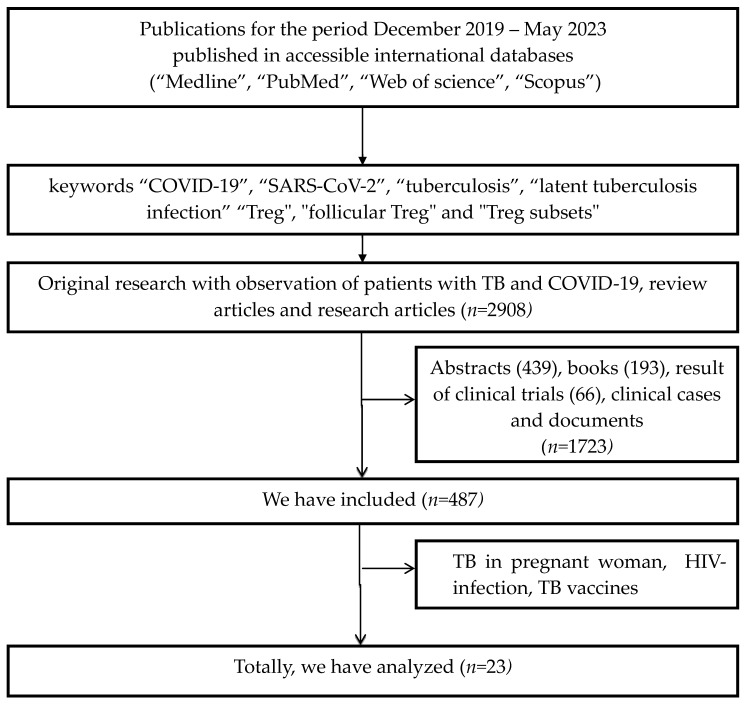
Flow chart depicting a systematic literature search.

We analyzed publications, released from December 2019 to May 2023 after searching for works published in accessible international databases (“Medline”, “PubMed”, “Web of Science”, “Scopus”) with search parameters for keywords such as “COVID-19”, “SARS-CoV-2”, “tuberculosis”, “latent tuberculosis infection”, “pulmonary tuberculosis”, “autoimmune inflammation”, “Treg”, “follicular Treg”, and “Treg subsets”. Inclusion criteria were: Original studies with TB patients and COVID-19, reviews, and research articles. Exclusion criteria were: Clinical cases, abstracts, clinical trials, books, TB in pregnant women, HIV-infection, and TB vaccines. We analyzed 2908 publications excluding abstracts (439), books (193), results of clinical trials (66), and clinical cases and documents (n = 1723). We included 487 publications, analyzing a total of 23 publications (Figure 1).

The narrative review was carried out in accordance with the PRISMA protocol (http://www.prisma-statement.org, 6 March 2023 (registration ID 420213). The JBI checklist was used to evaluate the quality of the study.

## 3. Results

### 3.1. COVID-19 Affecting Immune Response in LTBI and Active Tuberculosis

According to the data analysis, a whole body of publications (n = 2908) related to COVID-19 affecting TB course were identified. However, while analyzing the publications in detail by taking into account the exclusion criteria, only 23 publications were selected, which contained the information on the published data and relevant results. The results of immunological studies underlying COVID-19-related effect on TB development and course are presented in Table 1.

According to the data presented in Table 1, four publications provided comparable data, regarding decreased CD4 T cell level in LTBI. At the onset of the COVID-19 pandemic, Rajamanickam A (2021) analyzed a wide range of immunological parameters in LTBI subjects [25], finding significantly different plasma levels for IFN γ, IL-2, TNF α, IL-1 α, IL-1 β, IL-6, IL-12, IL-15, IL-17, IL-3, GM-CSF, IL-10, IL-25, IL-33, CCL3, and CXCL10 in LTI along with COVID-19. A 2022 study by Petrone L et al. in LTBI during COVID-19 revealed a decline in CD8+ T cell count and IFN-γ level [28], accounting for a decreased immune response in LTBI. Such alterations may suggest a negative QFT Plus Gold test data in COVID-19 convalescents with LTBI. Hence, it necessitates a demand to develop new methods for LTBI investigation by taking into account altered immune response in SARS-CoV-2-infected subjects.

Single studies analyzed immunological parameters in tuberculosis patients, suffering from COVID-19. According to the published data, COVID-19 infection potentiates a decline in IFN-γ level, as well as magnitude of SARS-CoV-2-specific CD4 T cells [28,29]. Most researchers conclude that immune response in LTBI and tuberculosis is clearly impaired, which may presumably result in developing active tuberculosis or severe TB course in COVID-19 convalescents [29,30,31]. However, it was noted to continue performing further studies. We also attempted to analyze immune response in TB patients and compare alterations in immunological parameters due to the SARS-CoV-2 virus.

### 3.2. Immunological Features in Patients in Tuberculosis

TB infection is characterized by a great variability observed at diverse disease stages ranging from premalignant to manifest disease. It is accounted for by pathogen virulence and quantity upon body invasion, duration of contact, routes of infection, as well as host immune state [32,33].

Currently, it is acknowledged that Th1 cells in *M. tuberculosis*-infected humans are considered key effector T-helper cells. Upon recognition of the *M. tuberculosis*-specific antigen in peripheral tissues, Th1 cells begin to produce IFN-γ by activating a broad spectrum of immunocompetent cells, including cytotoxic CD8+ T cells, ILC1, macrophages, and B cells, which are involved in elimination of pathogens localized within host cells [34].

After *M. tuberculosis* infection, the first stage in developing tuberculosis is presented as a latent tuberculosis infection [35,36,37]. The major criteria for LTBI diagnostics include positive in vitro or in vivo immunological tests along with the lack of criteria for active tuberculosis infection [37]. The methodology of in vitro immunological tests is based on assessing activation of CD4+ or CD8+ T cells by specific peptides ESAT-6 and SFP-10, as well as IFN-γ release by antigen-specific activated CD4+ T cells [36,37,38]. Previously, it was shown that COVID-19 represents one of the causes eliciting a decline in CD4+ and CD8+ T cells, as well as IFN-γ levels, which may affect lowered diagnostic efficacy for LTBI [39,40].

As stated before the COVID-19 pandemic, about 10% of individuals with LTBI may develop active tuberculosis [36,41] under unfavorable conditions, such as stress, comorbidities, immunosuppression, etc., affecting macro-organisms and resulting in some immunologic disorders. At present, it is unclear how such a situation may change after the exposure of *M. tuberculosis*-infected subjects to SARS-CoV-2. Moreover, no follow-up data are available yet.

The identification of peptides that bind to MHC I and II molecules is crucial for the activation of CD8+ and CD4+ T-lymphocytes. Recent studies revealed that the engagement of both helper T-lymphocyte (HTL) epitopes binding to MHC II molecule and cytotoxic T-lymphocyte (CTL) epitopes interacting with MHC I molecule is required for mounting a strong immune response against *M. tuberculosis* (Mtb) [42].

Using TB human studies and experimental models enabled us to find out that lungs infected with Mtb contain organized ectopic lymphoid structures, bearing CXCR5+ T cells. In experimental models, the data uncovered that CXCR5 is expressed on T cells to ensure their proper localization within tuberculosis granulomas and promote effective macrophage activation to elicit a full defense against Mtb infection. Such data demonstrate that CD4+CXCR5+ T cells play a protective role in anti-tuberculosis immune response. According to the data obtained, the level of peripheral blood CXCR5-expressing Tfh cells in patients with active pulmonary tuberculosis is significantly lower than that in LTBI patients [41]. An imbalance in the Tfh subset profile is observed in tuberculosis, skewed towards a higher level of Tfh2 and Tfh17 cells, along with decreased Tfh1 cell level [43]. A similar pattern is also found in patients with autoimmune pathology [44]. Some studies have shown that Th17 cells play an important role in the production of cytokines, such as IL-23 and IL-17, that are necessary for the formation of tertiary lymphoid structures [45,46].

Increased IL-17 and IL-23, along with CXCL13 expression, has been verified in animal models, which provided CXCR5+ recruitment of Tfh and B cells into tertiary lymphoid structures. This event underlies an emergence of humoral immune response [47]. The role of Th17 cells in tuberculosis remains debated. It is known that some polymorphisms in the genes encoding signature Th17-associated cytokines, IL-17A and IL-17A, may be associated with a predisposition to tuberculosis [48]. It was noted that a decreased Th17 level is associated with a clinical cure for tuberculosis. At the same time, the suppression of Th17 cell pool due to lowered IL-6R expression may represent a crucial mechanism in the regulation of developing active TB [49]. It has been shown that the Th17.1 cell subset level is decreased in TB patients vs the control group, whereas peripheral blood “classic” and CCR6+DP Th17 cell levels are significantly elevated [50]. According to some data, an increased IFN-γ+IL-17+ T cell count correlated with disease severity. High levels of CD4+IFN-γ+IL-17+ T cells were most often detected in TB patients with low therapeutic efficacy and widespread lung damage, as well as prolonged disease course, which is related to impaired immune response [51,52]. Finally, animal models uncovered that IFN-γ-secreting Th17 lymphocytes negatively affect the development of long-term immune defense upon reinfection with *M. tuberculosis* [53,54].

According to studies, tuberculosis patients have a decreased level of memory B-cells and increased count of “naive” B-cells, compared to healthy subjects [55,56,57]. However, B-cell alterations have not been observed in other lung diseases, such as viral or bacterial pneumonia, bronchiectasis lung disease, bronchial asthma, or COPD [58]. Regulatory B-cells producing IL-10 and IL-35 were found in TB granulomas [57]. A subset of immature transient CD24++CD38+ B-cells performs diverse regulatory functions, the elevation of which was detected in tuberculosis patients compared to healthy subjects. CD5+ B-cells are also defined as IL-10-producing regulatory subset additionally involved in the synthesis of low-affinity autoreactive IgM antibodies [59]. 

The mouse tuberculosis model revealed an increased count of IL-35- and IL-10-co-producing regulatory B-cells. In turn, IL-35 can contribute to IL-35+ B-cell infiltration into lung tissue and suppress recruitment of tuberculosis-specific effector Th1/Th17 cells. An increase in the latter led to increased Foxp3+ Treg level in the site of lung tissue infiltration [57,60]. In addition, the level of Foxp3+ Treg cells differed significantly, in parallel with mycobacteria sputum microscopy data [53,54].

Peripheral blood Treg cells were more frequently found in patients with active vs. latent pulmonary tuberculosis and healthy subjects [61]. Mouse models demonstrated that the effect of Tregs in tuberculosis is related to the phase of inflammation so that, at a high level, they are observed in the acute phase of the disease, being associated with poor efficacy of disease treatment. A significantly higher percentage of Tregs was found in patients with drug-sensitive vs. multidrug-resistant TB patients. Studies showed that high Treg levels downmodulated PBMC-related microbiologic activity in Tbc patients [62]. Treg levels were significantly higher in BAL vs. peripheral blood [63].

CD127, an α-chain IL-7 receptor, is used to identify Tregs among CD4+CD25+ T cells. FoxP3, a signature marker of regulatory T lymphocytes interacts with the CD127 gene promoter and to suppress it [64]. CD39hi Tregs produce more IL-10 and exert stronger suppressor activity [65]. It has been shown that activation markers are expressed on Treg surface in patients with tuberculosis. The predominant Treg subset in both LNMC and PBMC from TB patients co-expresses HLA-DR and CD38 [66].

The presented data characterize the immune response mounted by *Mycobacterium tuberculosis* prior to the emergence of SARS-CoV-2 in global medical practice. Clearly, exposure to a single causative agent, resulting in a very specific bacterial infection, accompanied by an immunosuppressive response, along with autoimmune inflammation. It may aggravate immunocompromised condition after infection with a viral agent. The occurrence of such disorders can simultaneously alter the course of TB infection similar to co-infection with HIV [67]. The goal of our study in the context of the limited number of available studies and currently accumulated experience was to identify potential alterations, while comparing the data published on tuberculosis and COVID-19 co-infection. 

### 3.3. Immune Response in COVID-19 Patients

SARS-CoV-2 directly affects airway tract cells, mediated by circulatory disorders along with profoundly altered immune response in both severe and mild COVID-19 [12,13,19].

SARS-CoV-2 causes the dysregulation of innate immunity with increased peripheral blood neutrophil levels that promote de novo formation of neutrophil extracellular traps (NETs) [68,69,70]. The emergence of tissue NET components, such as proteases, DNA degradation products, and neutrophil histones, as well as high levels of IL-6 and IL-17A, support a vicious circle of hyperinflammation, endothelial damage, thrombosis, and cardiovascular dysfunction [71,72].

Lymphopenia has been described in many patients with COVID-19, primarily characterized by lowered CD4+ and CD8+ T-cell counts also observed in several coronavirus infections [28]. SARS-CoV-2-specific CD4+ T cells express IFN-γ, TNF-ά, and IL-2, evidencing about developing Th1-type cell response [73]. An important role of CD4+ T-cells has been shown in murine models, wherein reduced T-lymphocyte count promoted the emergence of more prominent pulmonary inflammation. Transfer of SARS-CoV-2-specific CD4+ and CD8+ T cells to immunodeficient mice also resulted in mounting an effective immune response to the mouse-adapted SARS-CoV-2 strain [26]. CD8+ T cells were shown to be critical for controlling SARS-CoV-2 infection during the acute phase of COVID-19 and altered CD8+ T cell subset composition, dramatically influencing an efficient antiviral immune response [74,75]. 

A decrease in circulating follicular Treg cell count was noted in peripheral blood of acute COVID-19 patients compared to control group [76]. Moreover, Zahran et al. obtained similar data, showing that hospitalized severe patients had lower level of peripheral blood CD4+CXCR5+ICOS+Foxp3+ Tfr cells [77,78]. It should be noted that a consistently low level of circulating CD45RA-CD127-CD25+CXCR5hiPD-1h Tfr was also observed in COVID-19 patients [79]. 

As previously mentioned, the most important phenotypic characteristic of Th1 cells is that they express CXCR3 on the surface of the chemokine receptor, allowing it to enter the foci of inflammation along the gradient of relevant chemokines—CXCL9, CXCL10, and CXCL11 [80,81]. Patients with severe COVID-19 had increased serum levels of CXCR9 and CXCR10, which, along with the elevated count of both cellular (“unclassified” monocytes, CD38+HLA-DR+ T-cells and granzyme-B+/perforin+ T-cells) and serum (CXCL8, IL-6 and IL-10 levels) cues, allowed us to differentiate mild from severe disease [82]. In addition, COVID-19 convalescent patients showed a negative correlation between levels of circulating Tfr cells and serum virus-specific IgM, IgG, and IgA antibodies. Follicular Treg cells may potentially play an important role in controlling humoral memory response and antibody specificity, as well as preventing auto-antibody formation [83].

COVID-19 is also featured with decreased percentage of T-helper cells bearing key surface Th17 cell markers—CD161 and CCR6—compared to control group [84]. Th17 cells during COVID-19 are characterized by phenotype typical to tissue resident memory T cells also expressing the genes associated with cytolytic potential (SRGN, GZMB, and GNLY) and cytokine genes encoding IL-21, IL-17F, IL-17A, IFN-γ, and GM-CSF. Moreover, lung tissues collected from COVID-19 patients were enriched in CCR6 and IL-17A co-expressing cells [85]. Thus, patients with acute COVID-19 infection are characterized by decreased Tfr, along with elevated Tfh levels, that were able to contribute to mounting humoral autoimmune responses and the emergence of autoimmune pathologies in post-COVID-19 syndrome [86,87,88].

The aberrant organization of B-dependent zones, resulting in poor humoral immune response in patients with severe acute COVID-19, was noted in numerous studies [89]. The number of follicular dendritic cells, Bcl-6+ Tfh, and B-cells decreased in lymph nodes, whereas AID+ B-cell level was preserved most often [90]. The rise of the latter in cases of severe COVID-19 may suggest things about immunosuppression and the spread of virus-related pathological effects. CD25 expression was increased on Treg cells in patients with severe COVID-19, which was partially reduced after recovery that, however, remained elevated compared with healthy subjects. A unique type of hyperinflammatory response against SARS-CoV-2 may develop in some patients, eliciting autoimmune reactions. One of the plausible causes, resulting in development of autoimmune complications, may be due to molecular mimicry between SARS-CoV-2 S proteins and human proteins [28]. At the same time, the course of COVID-19 infection can be markedly affected, not only by host genetic predisposition, but also by underlying lung diseases. In this regard, the investigation of various types COVID-19 course in tuberculosis patients is of particular interest [23,80].

Thus, severe acute COVID-19 was found to have frequent compromised adaptive immune response due to the decline of the T-helper cell arm and reduced B-cell-dependent zones in secondary lymphoid organs. However, primarily enhanced activity of the above-mentioned cell types may develop allergic or autoimmune pathology during the recovery period.

### 3.4. Comparison of M. tuberculosis and SARS-CoV-2-Triggered Immune Response

Clearly, the interplay and impact of these two distinct pathogens on the development and course of tuberculosis infection have been discussed. Prior to the COVID-19 pandemic, more relevant insights into tuberculosis/HIV co-infection have been obtained. Now, it is clear that the effect on SARS-CoV-2-related immune response is not limited to a single COVID-19 episode. SARS-CoV-2 exerts long-lasting effects on the human immune system not always driven by COVID-19 severity. Tuberculosis infection is caused by *M. tuberculosis* that may proceed as a latent or active tuberculosis capable of resulting in generalization, low therapeutic efficiency, and development of drug resistance. Altogether, such events not only result from pathogen-related characteristics, but also due to features of host immune response. Not only the course of tuberculosis process per se, but also a crosstalk with a pathogen, such as SARS-CoV-2, are shaped by immune response parameters related to individualized genetic background. Circulating Tfh cells are distinguished from other Th cell subsets by CXCR5 chemokine receptor expression [59] also expressing chemokine receptors specific to polarized non-follicular Th cell subsets, such as Th1, Th2, and Th17 [60]. Regulatory T cells also play a crucial role in controlling chronicity of inflammatory reactions. An immune response triggered in COVID-19 and Tbc patients is characterized below (Table 2).

According to the current data, both tuberculosis and COVID-19 patients show elevated Th1 and Th2 cell levels typical to activated immune response. More often, tuberculosis is associated with a decline in Th17 cell level that may be further exacerbated by COVID-19. The typical course of tuberculosis is coupled to increased Treg cell level, whereas COVID-19, along with Tbc, results in a hyperinflammatory response. A shift to the latter may prevent virus spread while exacerbating the course of TB infection [108,111]. However, no unequivocal data on long-term data related to treatment and the course of tuberculosis in COVID-19 convalescent patients are available [112]. Currently, published study data suggest the need to further evaluate and monitor patients with high risk of active tuberculosis by taking into consideration development of post-COVID-19 immunosuppression.

## 4. Discussion

It is recognized that Th17 cells are actively involved in the pathogenesis of multiple pro-inflammatory events in both autoimmune and infectious disorders. The cytokines IL-1β, IL-6, and IL-23 play a crucial role in Th0-to-Th17 cell “polarization” [53,54].

COVID-19 is characterized by a decreased percentage of T-helper cells bearing the key Th17 surface markers, CD161 and CCR6 [91]. A similar pattern was found by using diverse biological research approaches. It was shown that the expression of Th17 cell-associated genes (RORC, IL17A, IL17F, and CCR6) was downmodulated in peripheral blood CD4+ T cells in COVID-19 patients [80]. Such cells migrated to the foci of inflammation that was confirmed by BAL studies. During COVID-19 infection, BAL Th17 cells had phenotype of tissue resident memory T cells and also expressed genes associated with cytolytic properties (SRGN, GZMB, and GNLY), and cytokine genes (IL-21, IL-17F, IL -17A, IFN-γ, and GM-CSF). Moreover, the lung tissues in COVID-19 patients were enriched in CCR6- and IL17A-co-expressing cells. The high concentration of IL-6, IL-17A, GM-CSF, and IFN-γ were found in the BAL liquid phase which may account for the volumetric inflammatory changes in severe patients with pneumonia [14,95]. IL-17A was shown to be required for granuloma maturation during mycobacterial infections [54]. 

Circulating human Tfh cells can be divided into four subsets based on the expression of two chemokine receptors, CXCR3 and CCR6. Thus, the Tfh1 population are phenotyped as CXCR3+CCR6−, Tfh2—CXCR3−CCR6−, Tfh17—CXCR3−CCR6+, as well as CXCR3+CCR6+ “double-positive” DP Tfh or Tfh17.1 cells, which differ in related functional properties. Hence, Tfh2 and Tfh17 cells (CXCR3–CCR6– and CXCR3–CCR6+, respectively) are able to promote antibody class switch in the “naive” B cells they synthesize antibodies and secrete (from IgM to IgG and IgE or IgG and IgA, respectively), whereas CXCR3+ Tfh1 memory cells are unable to exert such a stimulating effect, and can induce apoptosis in activated “naive” B-lymphocytes [94].

Peripheral blood CD8+ T cells can be subdivided into Tc1, Tc2, and Tc17 subsets based on surface chemokine receptor expression. Moreover, each type of such cells plays a distinct role in inflammation, both in vivo and in vitro. Few studies on this type of CD8+ T cell subset have been published. However, researchers who were engaged in this field obtained a rather complete picture for pathogenesis of certain pathologies. At the same time, the vast majority of Tc17 cells display a terminally differentiated phenotype (CD27-/CD45RO-) and profoundly suppress Th1 and Th17 cell functional activity evidencing their potent in relation to anti-inflammatory effects [95]. 

We also consider the importance of evaluating a pool of T-regulatory cells firstly by distinguishing based on surface expression of chemokine receptors, CCR4, CCR6, and CXCR3, enabling the indirect assessment of the migration of such cells into the lung tissue. In the context of the above pathologies, no studies have been conducted yet. According to the chemokine receptors noted above, regulatory T cells are subdivided into CCR4+CCR6–CXCR3+ Th1-like (Tr1), CCR4+CCR6–CXCR3- Th2-like (Tr2), CCR4+CCR6+CXCR3- Th17-like (Tr17), and CCR4+CCR6+CXCR3+ Th17.1-like (Tr17.1) cell subsets. The Th2-like Tregs display higher survival and migratory potential, as well as more prominent selectivity for T-effector cell suppression [96,113]. Th1-like T-regulatory cells and Th17-like T-regulatory cells exert increased pro-inflammatory activity by secreting IFN-γ and IL-17, respectively. However, they can also secrete IL-10, but at a lower level [97]. The former have the CD4+Foxp3+CD25+CD31+Helios+Nrp1+ phenotype, whereas the latter have the CD4+Foxp3+CD25+CD31-Helios-Nrp1 expression pattern [6]. Recent peripheral blood studies revealed that the prevalence of CD4+CD25+ T cells was higher in patients with active Tcb vs. LTBI. However, no differences in the number of CD4+CD25+CD127lo, CD4+CD25+FoxP3+, or CD4+CD25+FoxP3+CD127lo T cells were found [103]. 

Severe COVID-19, similarly to tuberculosis, was characterized by increased Th cell count, which may be related to immunosuppression. CD25 expression was upregulated on regulatory T cells in severe COVID-19 that was partially decreased after recovery but remained higher than in the control group. CD127 expression was evenly downmodulated in both mild and severe COVID-19 patients compared with healthy subjects. At the same time, after recovery, its expression returned back to normal level solely in patients with the mild disease, while continued to decrease in severe COVID-19 [14]. In addition, it was also shown that all COVID-19 convalescent vs. healthy subjects have low levels of CD25+Foxp3+CD127- regulatory T cells [26,59]. Kalfaoglu et al. found a decline in FOXP3+IL2RA+CD4+ T-cell levels during severe vs. moderate COVID-19 [91]. This immune response coupled to emerging post-COVID-19 immunosuppression in patients with tuberculosis may profoundly impact on further progression of the pathology [103,113].

**Figure 2:**
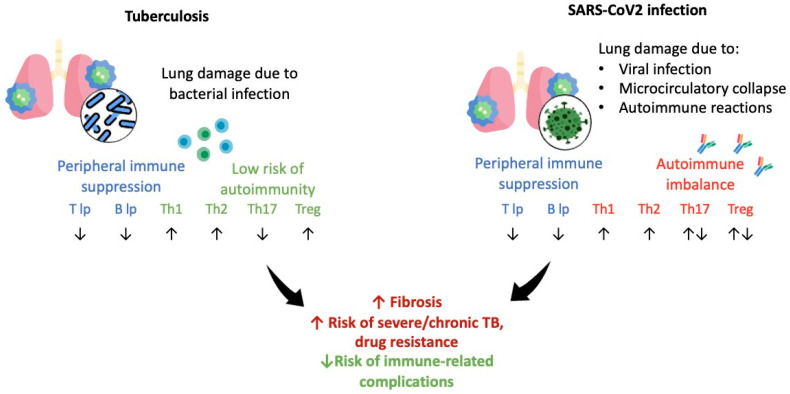
The scheme depicting Th cell immune response altered in tuberculosis and COVID-19, ↑—high level; ↓—low level.

The alterations in cell type profile during tuberculosis and COVID-19 are shown in Figure 2. 

Antigen-specific peripheral tissue effector Th1 cells can produce IFN-γ to activate a broad range of immunocompetent cells, including CD8+ T cells, ILC1, macrophages, and B-cells, which are involved in intracellular pathogen elimination [80,114].

IFN-γ and TNF-α overproduced by Th1 cells during response to SARS-CoV-2 elicit a mass death of virus-infected cells resulting in lung tissue damage and triggering acute respiratory distress syndrome [113,114,115]. Hence, Th1 cell migration to inflamed tissues barely accounts for a decline in some fraction of such peripheral blood cells during acute TB infection that was noted in several independent studies [105,108,116,117].

## 5. Conclusions

Current data on the alterations in immune response during LTBI and tuberculosis after COVID-19 remain insufficient. Numerous conflicting clinical data suggest a decreased pulmonary function in TB patients and an increased risk of adverse COVID-19 outcomes. To some extent, immune responses in COVID-19 and TB infection overlap ranging from imbalanced Th cell subset composition, pro-inflammatory cytokine production to altered B-cell activation, and excessive infiltration of inflammatory sites by highly activated peripheral blood cells. It may result in excessive tissue damage. Both SARS-CoV-2 and Mtb lead to imbalanced and dysregulated immune response that elevates a risk of developing TB infection and its severe course. An introduction of such findings may improve the future management of individuals with LTBI and tuberculosis. Therefore, identification of novel immunological features for tuberculosis during or after COVID-19 will provide a deeper insights into diagnostics and treatment of relevant pathological conditions.

## Figures and Tables

**Table 1:** COVID-19 affects immune response and development of tuberculosis infection.

TB Infection and COVID-19	Immune Response	First Author, Year of Publication
LTBI + COVID-19 (clinical case)	decline in CD4+ T cell count along with latent-to-active TB progression	Khayat M, 2021 [24]
LTBI + COVID-19 (study data)	LTBI with COVID-19 showed significantly higher plasma levels of IFN γ, IL-2, TNF α, IL- 1 α, IL-1 β, IL-6, IL-12, IL-15, IL-17, IL-3, GM-CSF, IL-10, IL-25, IL-33, CCL3 and CXCL10	Rajamanickam A, 2021 [25]
LTBI + COVID-19 (review)	decreased cytokine levels shown for IL-2, IL-4, IL-5 and IL-13	Shariq M, 2022 [26]
LTBI + COVID-19 (study data)	LTBI in COVID subjects had lowered CD8+ T cell count and IFN-γ level	Palacios-Gutiérrez JJ, 2022 [27]
TB +COVID-19 (study data)	low level of IFN-γ in COVID-19 and TB patients	Petrone L, 2021 [28]
TB +COVID-19 (study data)	decreased SARS-CoV-2-specific CD4 T cell level in COVID-19 and TB patients	Flores-Lovon K, 2022 [29]

**Table 2:** Th cell subset alterations in tuberculosis and COVID-19.

Cells	COVID-19	Tuberculosis
Th1	↑ [65,91,92]; ↓ [93,94]	↑ [95,96]
Th2	↑ [97,98,99]	↑ [100,101]; not significant [95]
Th17	↑ [99,102]; ↓ [93,96,101]	↓ [100,103]
Tfh	↑ [98,104]; ↓ [87,91,99,105]	↑ [100]; ↓ [101] not significant [95]
Treg	↑ [65,106,107]; ↓ [66,108,109]	↑ [43,60,110,111,112]

↑—high level; ↓—low level.
